# White matter microstructure alterations from alcohol use disorder persist into early abstinence

**DOI:** 10.1093/braincomms/fcag018

**Published:** 2026-01-20

**Authors:** Nicolas Delinte, Melissa Salavrakos, Manon Dausort, Laurence Dricot, Pauline Hermans, Philippe de Timary, Benoit Macq

**Affiliations:** Institute of Information and Communication Technologies, Electronics and Applied Mathematics (ICTEAM), UCLouvain, Louvain-la-Neuve 1348, Belgium; Institute of NeuroScience (IoNS), UCLouvain, Brussels 1200, Belgium; Institute of NeuroScience (IoNS), UCLouvain, Brussels 1200, Belgium; Institute of Information and Communication Technologies, Electronics and Applied Mathematics (ICTEAM), UCLouvain, Louvain-la-Neuve 1348, Belgium; Institute of NeuroScience (IoNS), UCLouvain, Brussels 1200, Belgium; Institute of Information and Communication Technologies, Electronics and Applied Mathematics (ICTEAM), UCLouvain, Louvain-la-Neuve 1348, Belgium; Institute of NeuroScience (IoNS), UCLouvain, Brussels 1200, Belgium; Institute of Information and Communication Technologies, Electronics and Applied Mathematics (ICTEAM), UCLouvain, Louvain-la-Neuve 1348, Belgium

**Keywords:** white matter, Alcohol Use Disorder, multi-fixel, microstructure, multi-shell

## Abstract

Alcohol use disorder (AUD) is a complex condition including affective, cognitive and motivational dimensions. Although AUD is known to induce diffuse brain damage, including grey matter shrinkage and ventricular enlargement, the microstructural changes it induces in white matter remain incompletely understood. This study leverages multi-shell diffusion MRI and multi-fixel models to (i) undertake whole-brain and tract-specific analyses to investigate the microstructure of white matter (WM) tracts affected by AUD, (ii) evaluate whether these differences persist in early abstinence, and (iii) correlate these results with clinical measures evaluated by validated psychological questionnaires. We recruited a final cohort of 37 AUD patients, admitted for alcohol withdrawal and selected for their ongoing alcohol consumption at the time of admission, and a demographically matched control group of 19 healthy subjects. Both groups underwent MRI scans at baseline and 18 days later, with assessments of depression, obsession-compulsion, and anxiety conducted in both sessions for the AUD patients and once for the control group. The imaging results confirmed the presence in AUD participants of clusters microstructural alterations in the fornix, corpus callosum, cingulum, uncinate fasciculus and anterior thalamic radiations. These white matter tracts presented global and localized microstructural changes in axial diffusivity and fractional anisotropy, which are linked to axonal damage and inflammation. There was no significant improvement in the diffusion metrics after almost three weeks of abstinence, although clinical measures did improve significantly. Depression scores were significantly elevated in the patients at admission and decreased with time. Depression scores before withdrawal showed correlations with microstructural metrics across the right anterior thalamic radiations, the isthmus of the corpus callosum, and the right uncinate fasciculus. Lower fractional anisotropy and higher radial diffusivity were predictive of higher depression scores. Overall, these findings highlight the long-term vulnerability of WM tracts affected by AUD and the link between tract microstructure, brain function and behaviour.

## Teaser

Using multi-shell diffusion MRI and multi-fixel models, Delinte *et al*. identify persistent microstructural alterations in key white matter tracts in individuals with alcohol use disorder. These alterations did not recover during early abstinence and are related to depressive symptoms, highlighting the enduring impact of alcohol use on brain microstructure.

## Graphical Abstract

**Graphical Abstract:**
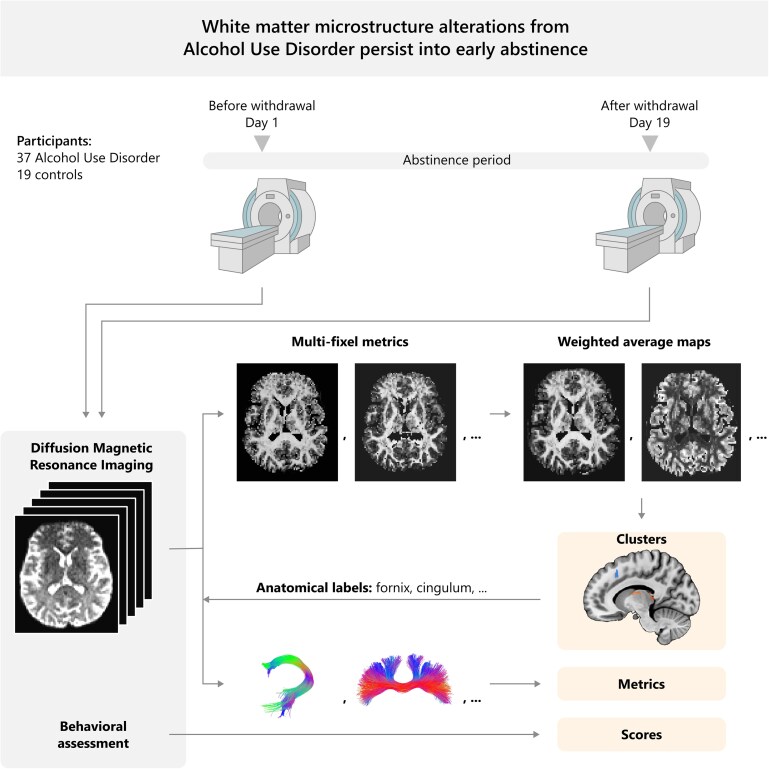

## Introduction

Alcohol Use Disorder (AUD) is a condition characterized by a persistent pattern of excessive alcohol consumption, where individuals present a loss of control over their alcohol intake despite the negative consequences on mental and physical health, social and work obligations, and relationships.^1^ As a multifaceted disorder encompassing affective, cognitive and motivational dimensions, AUD represents a significant public health concern.^2,3^ One of its pathological mechanisms involves the direct neurotoxic effects of alcohol, which induce astrocyte swelling, leading to oxidative and nitrosative stress, disrupting intracellular signalling pathways, and altering gene and protein expression.^4^ Among the brain structures affected by chronic alcohol use, the white matter (WM) tracts appear particularly vulnerable. Damage to WM is consistently reported in various types of substance dependence^5^ and is believed to underlie many of the cognitive and executive deficits observed in AUD. However, accurately characterizing WM alterations is a major challenge due to the complexity of its microstructural organization. While conventional magnetic resonance imaging (MRI) techniques have highlighted macrostructural damage in the brain—such as grey matter volume loss,^6,7^ ventricular enlargement,^8,9^ and global white matter deterioration^5,10^—such methods often lack the resolution needed to capture more subtle microstructural changes.

The development of diffusion-weighted MRI (dMRI) sequences helped provide insight into the WM microstructural changes occurring in AUD. These microstructural properties are commonly indexed by diffusion metrics such as fractional anisotropy (FA), mean diffusivity (MD), axial diffusivity (AD), and radial diffusivity (RD), which reflect fibre coherence, overall water mobility, axonal integrity, and myelin integrity.^11^ Findings of altered structural connectivity, i.e. a reduction in FA alongside increases in AD, RD and MD in prefrontal WM pathways may underlie the deficits in executive performance in treatment-seeking AUD.^12^ Other dMRI studies also revealed significantly reduced FA and elevated MD in cortico-striatal fibres, frontal WM, and limbic pathways in treatment-seeking individuals early in abstinence, suggesting that disruption of these circuits may underlie deficits in executive function and impulse control.^13,14^ Chronic alcohol exposure also alters microstructure in key limbic WM tracts—specifically, the cingulum shows decreased FA and increased MD in frontal and parahippocampal segments,^12,15,16^ while the uncinate fasciculus exhibits decreased FA in orbitofrontal–temporal connectivity.^17^ The latter study also linked a loss of microstructural integrity in the fimbria to cognitive impairments in AUD subjects.^17^ Furthermore, individuals exhibiting lower FA and higher MD in the corpus callosum and fornix showed higher relapse rates.^18^ Finally, increased FA alongside decreased MD and RD in the corpus callosum and the internal capsule of AUD patients were correlated with the occurrence of panic disorder as a comorbidity.^19^

In 2022, a meta-analysis by Spindler *et al*.^20^ pooled the results from 18 different studies examining WM changes through voxel-based morphometry (VBM) and diffusion tensor imaging (DTI). The compiled literature revealed significant clusters, i.e. spatially contiguous groups of brain voxels of convergent macro- and micro-structural WM alterations compared to healthy controls. These clusters included the genu and body of the corpus callosum, the fornix, the anterior and posterior cingulum bundle, as well as the right posterior limb of the internal capsule. Their meta-analysis based on DTI studies only identified an additional cluster in the posterior parts of the left corpus callosum. However, studies in the meta-analysis differed with respect to patient populations, examining either actively drinking patients, patients undergoing withdrawal or those who were recently abstinent.

In their meta-analysis, Spindler *et al*. highlighted the importance of studies designed to unravel the respective clinical implications of the different clusters of WM alterations, and to evaluate the extent of reversibility of these AUD-related WM changes. The present study was designed to investigate these associations in a cohort of 53 AUD patients, admitted at our university hospital for alcohol withdrawal, and selected for their ongoing alcohol consumption at the time of admission. The alterations of white matter tracts were examined on the day of admission, and correlated with self-report questionnaires of depression, anxiety and craving evaluated using validated questionnaires on the next day. The evolution of these alterations was then investigated after a supervised withdrawal period of 18 days. The results in the AUD group were compared with a control population of 20 healthy subjects who also underwent clinical testing and two dMRI scans with an 18-day interval. This unique protocol allowed us to compare a well-defined group of AUD patients—heavy compulsive drinkers whose last alcohol intake had occurred within 24 h prior to admission—while ensuring that they remained abstinent throughout the 18-day follow-up period.

Despite consistent evidence of WM alterations in AUD, prior studies have often relied on DTI,^21^ a method limited in its ability to characterize fibres in areas of crossing fascicles. As a result, microstructural changes in regions of complex fibre architecture may be mischaracterized or overlooked. Moreover, the use of tools such as Tract-Based Spatial Statistics (TBSS)^22^ may compromise spatial resolution due to the registration onto a FA skeleton, in contrast to analyses conducted in native space. Additionally, techniques such as VBM^23^ may blend adjacent tract information due to the smoothing step and the registration of misaligned brain structures resulting from brain deformations or individual differences in the cortical folding pattern. These limitations highlight the need for advanced diffusion imaging techniques capable of resolving complex fibre architecture while preserving anatomical specificity. In this study, the tracts of interest were identified through an exploratory whole-brain, voxel-wise analysis using microstructural maps derived from a multi-compartment model. This analysis revealed clusters exhibiting significant differences between the AUD and control groups. The WM tracts intersecting these clusters were subsequently selected as the tracts of interest, providing a basis for tract-specific microstructural analyses.

## Materials and methods

### Participants

Participants were recruited from a population of patients diagnosed with AUD and hospitalized at Cliniques Universitaires Saint-Luc for a three-week medically supervised alcohol withdrawal. All AUD participants were actively drinking until the day of admission; their last alcohol intake occurred the day of admission or the day before. Exclusion criteria included the presence of severe psychiatric comorbidities (i.e. schizophrenia or bipolar disorder) as estimated by a psychiatrist (MS, PdT), chronic inflammatory diseases (i.e. lupus or vasculitis), regular use of anti-inflammatory drugs, the presence of metallic implants (i.e. pacemakers) or unremovable jewellery causing artefacts in the MRI scans. Fifty-three patients met these criteria and were willing to participate in the study. The sample consisted of 42 men and 11 women, with a mean age of 47.64 years (SD:±10.5; minimum: 28, maximum: 71). Most patients were smokers (29 smokers/24 nonsmokers). Their average daily alcohol consumption was 17.34 units (SD:±11.35; 1 unit = 10 g of ethanol). The numerical difference in alcohol intake between men and women was not statistically significant (18.9 units ±11.7 for men vs 11.36 units ±7.67 for women, *F* = 2.192, *P-value* = 0.145). They underwent MRI scans on the day of admission (E1) and after 18 days of withdrawal (E2). In the initial first 7 days, participants received treatment with oral diazepam (a benzodiazepine) to prevent the emergence of severe withdrawal symptoms (i.e. seizures, confusion). The initial dose was 4 × 10 mg in the first 48 h and was progressively tapered thereafter. All patients also received thiamine supplementation as prophylaxis against Gayet Wernicke syndrome.

Additionally, a control group composed of 20 healthy adults was recruited for comparative analysis. They did not differ for age (mean age was 51.3 years ±15.07, *P* = 0.245) or gender (13 men/7 women, *P* = 0.233). The demographic information of both cohorts is summarized in Supplementary Table 1.

**Figure 1:**
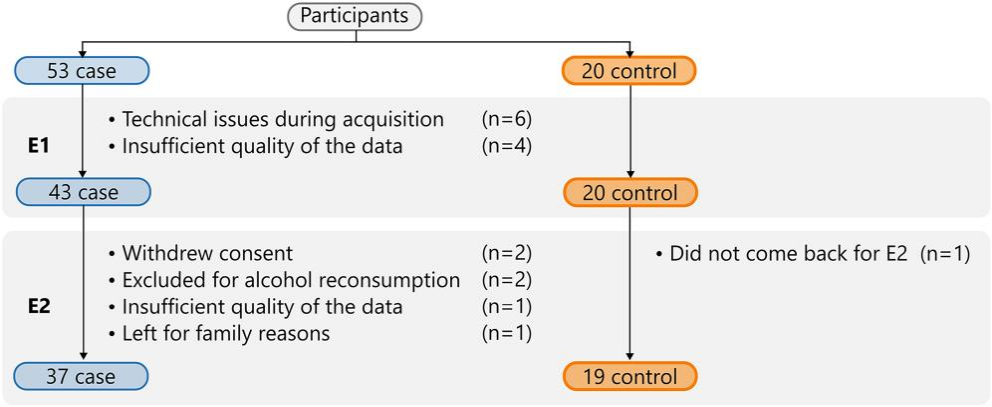
Representation of the participants in the case and control groups, as well as the respective dropouts in each cohort for each acquisition at admission (E1) and after the 18-day abstinence period (E2).

For six patients, technical problems occurred during the first MRI acquisition, such as excessive motion or the presence of metallic implants, leading to the creation of artefacts. Four more patients had insufficient data quality for the diffusion-weighted MRI sequence, including scans with high noise levels. This left a total of 43 patients for E1. Two patients withdrew their consent before E2, two others were excluded for alcohol reconsumption, and one patient had to leave the hospital for family reasons. One patient had the second MRI at E2 but the quality of the diffusion data was insufficient. One control subject also dropped out. After exclusions for motion, noise and dropout, 37 AUD and 19 control participants had interpretable data at both timepoints, see Fig. 1 for a summary of the dropouts.

The experiment was carried out in accordance with the ethical standards of the Declaration of Helsinki and received approval from the Ethics Committee of the University Hospital of Saint-Luc (number: B403201523514). Written informed consent to participate in this study was provided by the participant or the participants’ legal guardian/next of kin.

### Clinical assessment

**Figure 2:**
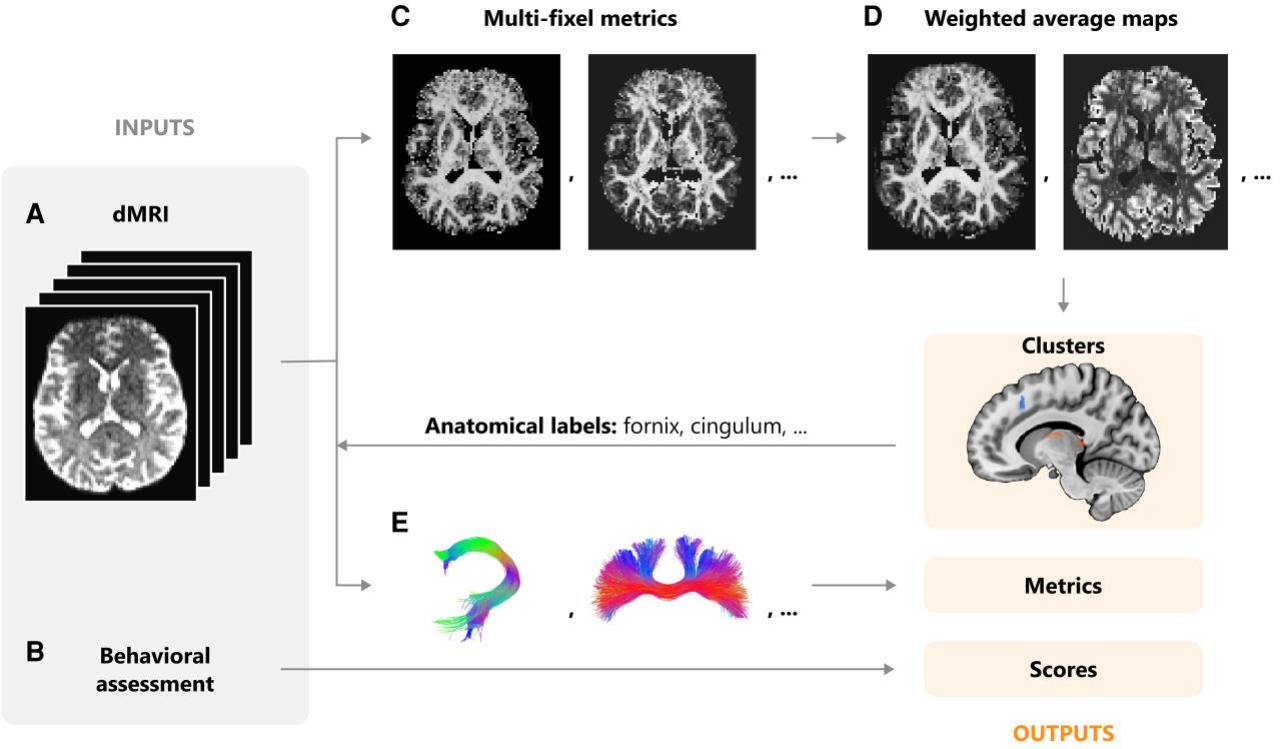
**Global overview of the analysis pipeline.** The two main inputs are: (**A**) preprocessed diffusion MRI data and (**B**) clinical assessments. The (**C**) metric outputs of the multi-fixel model are combined to create (**D**) whole-brain microstructure maps. The maps are used to detect clusters of interest. The locations of the clusters are used to select WM tracts of interest and (**E**) tractography is then generated to obtain tract-specific microstructural metrics.

On the 2nd and 18th day of the programme—within 24 h of the first and second MRI scans—clinical data was collected from all AUD participants using validated questionnaires, including the Beck Depression Inventory (BDI) for depression,^24^ the State Anxiety Inventory (SAI) for anxiety,^25^ and the Obsessive Compulsive Drinking Scale (OCDS) for obsessions and compulsions related to drinking^26^ (Fig. 2B). For the OCDS, a modified version was employed, excluding the items related to current alcohol consumption, as patients were undergoing detoxification and hence did not consume alcohol. Healthy controls underwent clinical assessment only once, at the same time as their first MRI scan.

### Data acquisition and pre-processing

The 37 AUD participants underwent two MRI scans: the first and second scans were performed on day 1 and 19 of the three-week withdrawal period. Similarly, the 19 healthy adults underwent two MRI scans 18 days apart. All scans were performed on a 3T GE SIGNA^TM^ Premier scanner (GE Healthcare, Chicago, IL). In each MRI acquisition, a 3D T1-weighted image was acquired with the following parameters: echo time (TE) = 2.96 ms, repetition time (TR) = 2188.16 ms, inversion time (TI) = 900 ms, 156 slices, 1 mm isotropic, in-plane field-of-view (FOV) = 256 × 256 mm^2^.

The dMRI scans were performed with the following parameters: TR = 4837 ms, TE = 80 ms, 2 mm isotropic voxels, in-plane FOV = 220 × 220 mm^2^, 110 × 110 matrix, 68 slices, 168 directions, Δ = 35.7 ms, δ = 22.9 ms, 64 gradients at *b* = 1000, 32 at *b* = 2000, 3000, 5000 s/mm^2^, corresponding to diffusion gradient intensities up to 68.9 mT/m, and 7 interspersed b0 images.

Preprocessing of the diffusion data was performed using the Elikopy pipeline,^27^ which included brain extraction,^28^ thermal denoising,^29^ eddy-current distortion and head-motion correction^30^ using FSL (v6.0.7.8).^31^ Synb0-DISCO^32^ was used to synthesize a distortion-free unweighted diffusion image from the T1-weighted image (Fig. 2A). The presence of gradient cycling in the sequence introduced slice-to-volume motion, which could not be addressed with the motion correction algorithm during preprocessing, and will be considered below in the metric analysis.

Noise and movement during the scan were estimated with QUAD.^33^ The scans exhibiting excessive motion, defined as an absolute displacement greater than 5 mm in any direction, were removed from the study (Fig. 1).

### Microstructural model

The present study addresses the limitations of prior dMRI approaches by leveraging multi-shell, high-gradient dMRI data, analysed with a multi-fixel model. Multi-fixel models estimate the properties of multiple fibre populations per voxel, known as *fixels*, enabling the investigation of microstructural metric evolution along specific tracts without considering the microstructural properties of crossing tracts.^34^

To evaluate the microstructural differences between both cohorts, DIAMOND^35^ was selected as the multi-fixel model. DIAMOND provided tensor-derived metrics, including FA, AD, RD and MD for each estimated fixel (Fig. 2C).

Two fibre populations and an isotropic signal contribution were allowed in each voxel, and each compartment was attributed a volume fraction. The model selected for the fascicles was ‘diamondcyl’. The diffusivity of the isotropic compartment was set to 3 × 10^−9^ *m*^2^/*s* to match the diffusion coefficient of free water at 37°C.^36^

### Whole-brain analysis

A whole-brain analysis was performed to identify tracts of interest and ensure that all significant WM regions were accounted for. The multi-fixel model generated a number of metric maps corresponding to the number of fixels *K*, with *K* = 2 in our case. These maps are affected by correspondence issues between adjacent fixels due to the absence of information about the macroscopic organization of the neural fibres.^34^ To bypass these correspondence issues in the whole-brain analysis, the per-fixel metrics *M*_*k*_ estimated by DIAMOND were aggregated into a single microstructure map per metric using the relative volume fraction weight for the mean voxel value.^34^ The weighted metric is defined as


(1) $$wM=\frac{\sum_{k=1}^{2}\;f_{k}M_{k}}{\sum_{k=1}^{2}\;f_{k}},$$

where *M* is the metric, *k* the fixel number and *f* its relative volume fraction.

Whole-brain volume-weighted microstructure maps were computed for each AUD and control subject at E1 and E2 (Fig. 2D). A voxel-based analysis was performed on all microstructure maps in the MNI template space. Registration to the standard space was conducted using a two-step process: first, a rigid registration aligned the diffusion-weighted MRI data to the T1-weighted MRI subject space. Next, a non-rigid transformation was applied to align the T1-weighted subject space with the MNI ICBM152 template space. This procedure used a Python implementation of the ANTS algorithm.^37^ We ran a single-factor analysis of variance (ANOVA), corrected for age, to compare the AUD and the control participants. A *P*-value of 0.0001 was selected for significance. The results were corrected for multiple comparisons using a cluster-size thresholding method implemented in BrainVoyager, based on Monte Carlo simulations that estimate the probability of obtaining clusters of different sizes by chance.^38^ According to this procedure, the minimum cluster size thresholds were set to 40, 17, 25, and 24 for FA, AD, RD, and MD, respectively.

The anatomical labels of the WM tracts corresponding to these clusters were identified using the JHU White-Matter Tractography Atlas^39^ and XTRACT HCP Probabilistic Tract Atlas.^40^

### Tracking of the neural pathways of interest

**Figure 3:**
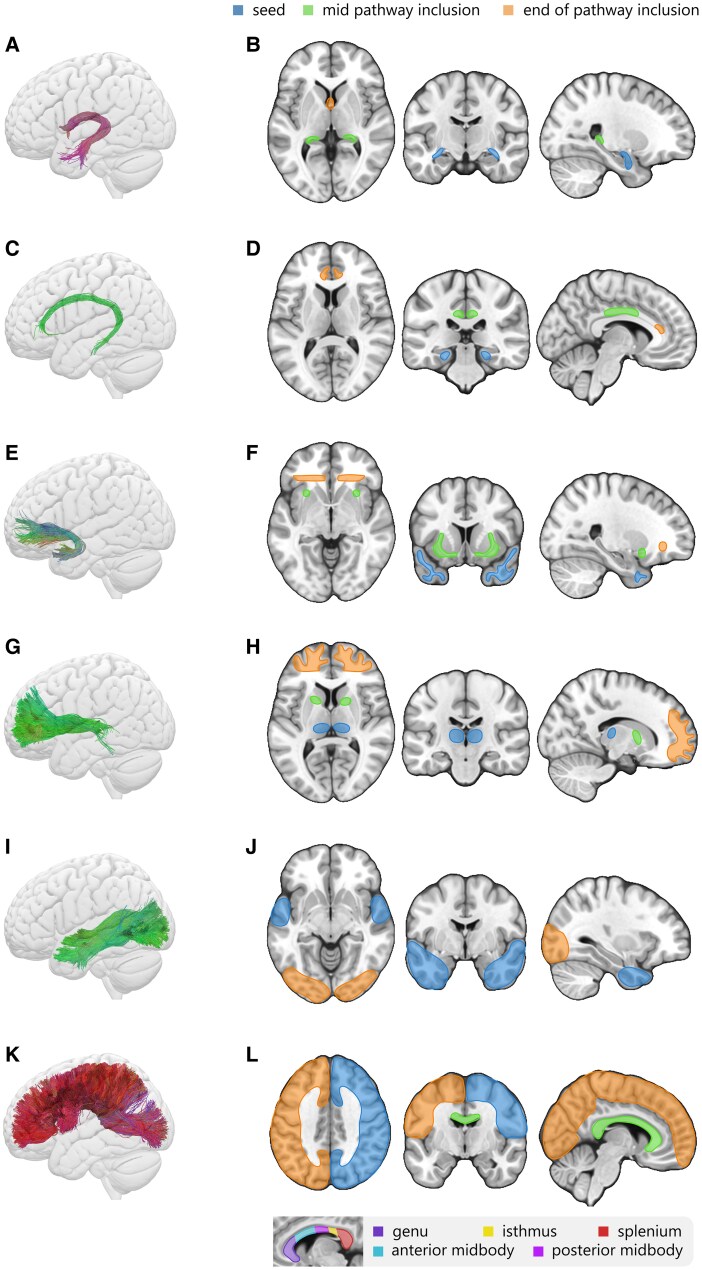
**Representation of the tracts of interest (A, C, E, G, I, K) and the corresponding seed (in blue) and inclusion (green, intermediary; orange, termination) regions used to generate the streamlines (B, D, F, H, J, L) of the fornix (A, B), cingulum (C, D), uncinate fasciculus (E, F), anterior thalamic radiations (G, H), inferior longitudinal fasciculus (I, J) and corpus callosum (K, L).** The tracts are colour-coded according to the average orientation of the streamline: red (left–right), green (anterior–posterior) and blue (inferior–superior).

Tractography was used to generate streamlines specific to the tracts of interest, based on the anatomical labels of the significant clusters obtained in the whole-brain analysis (Fig. 2E), namely the fornix, corpus callosum, cingulum, uncinate fasciculus, anterior thalamic radiations and inferior longitudinal fasciculus, illustrated in Fig. 3.

For local modelling, the MSMT-CSD algorithm^41^ was applied to make full use of the multi-shell data by exploiting the *b*-value dependencies of the different tissue types. Streamlines were obtained with the iFOD2^42^ algorithm available in the MRtrix3 software^43^ with the following parameters: maximum angle of 15°, step size of 1 mm, cut-off of 0.1 and the maximum number of streamlines per tract was set to 2000. The minimum and maximum length for each streamline were set to 10 and 200 mm, respectively. For each tract, 2 000 000 seeds were placed randomly within the seed inclusion regions displayed in Fig. 3. All regions of interest (ROIs) for the inclusion zones were drawn to have a width of at least 5 mm in the MNI space to ensure that all ROIs had a depth of at least two voxels when registered in the native diffusion space. The registration of the ROIs was performed using a Python implementation of the ANTS algorithm,^37^ which employed both linear and nonrigid transformations to optimize the mutual information between the FA maps on the patient’s native space and the FSL HCP1065 FA template. The output was cleaned by removing streamlines with isolated segments along their pathways based on kernel density estimates.^44^ The kernel density threshold was defined as five times the density of a single point, with a kernel bandwidth of 1.

The tracts and inclusion zones used to generate the streamlines are shown in Fig. 3 and detailed below:

**The fornix** (see Fig. 3A and B) is a bundle of neural fibres connecting the hypothalamus to several subcortical structures. The inclusion ROIs for the fornix were the columns (anterior pillars) and the crura (posterior pillars) of the fornix, and their connections to the hippocampus.^45^

**The cingulum** (see Fig. 3C and D) is a central structure that interconnects frontal, parietal and medial temporal brain regions, as well as subcortical nuclei to the cingulate gyrus. The ROIs used to generate the main pathway of the cingulum were the anterior part of the cingulum near the genu, and its midcingulate and parahippocampal portions.^46^

**The uncinate fasciculus (UF)** (see Fig. 3E and F) connects the orbitofrontal cortex to the anterior temporal lobes. Thus, inclusion ROIs were placed in the anterior temporal lobes, the external capsule and the extreme capsule, and a WM slice neighbouring the orbitofrontal cortex.^47^

**The anterior thalamic radiations (ATR)** (see Fig. 3G and H) are pathways connecting nuclear groups of the thalamus with the frontal lobe through the anterior limb of the internal capsule.^48^ The inclusion ROIs were the thalamus, anterior limb of the internal capsule and the WM neighbouring the prefrontal cortex.^49,50^

**The inferior longitudinal fasciculus (ILF)** (see Fig. 3I and J) is a long-range pathway connecting the occipital areas of the brain to the anterior temporal areas.^51^ The inclusion ROIs were the occipital and temporal lobes, dilated to include the adjacent WM.^50^

**The Corpus Callosum (CC)** (see Fig. 3K and L) is the main interhemispheric WM tract. The ROIs used for the isolation of the corpus callosum tracts were the left and right cortex, as well as the corpus callosum proper. To enable a more precise analysis of the parts of the corpus callosum impacted by AUD, the corpus callosum was further divided into five subsections (displayed in the sagittal view on Fig. 3L) using Hofer & Frahm’s scheme^52^: the genu, anterior midbody, posterior midbody, isthmus and splenium.

### Tract microstructure analysis

**Figure 4:**
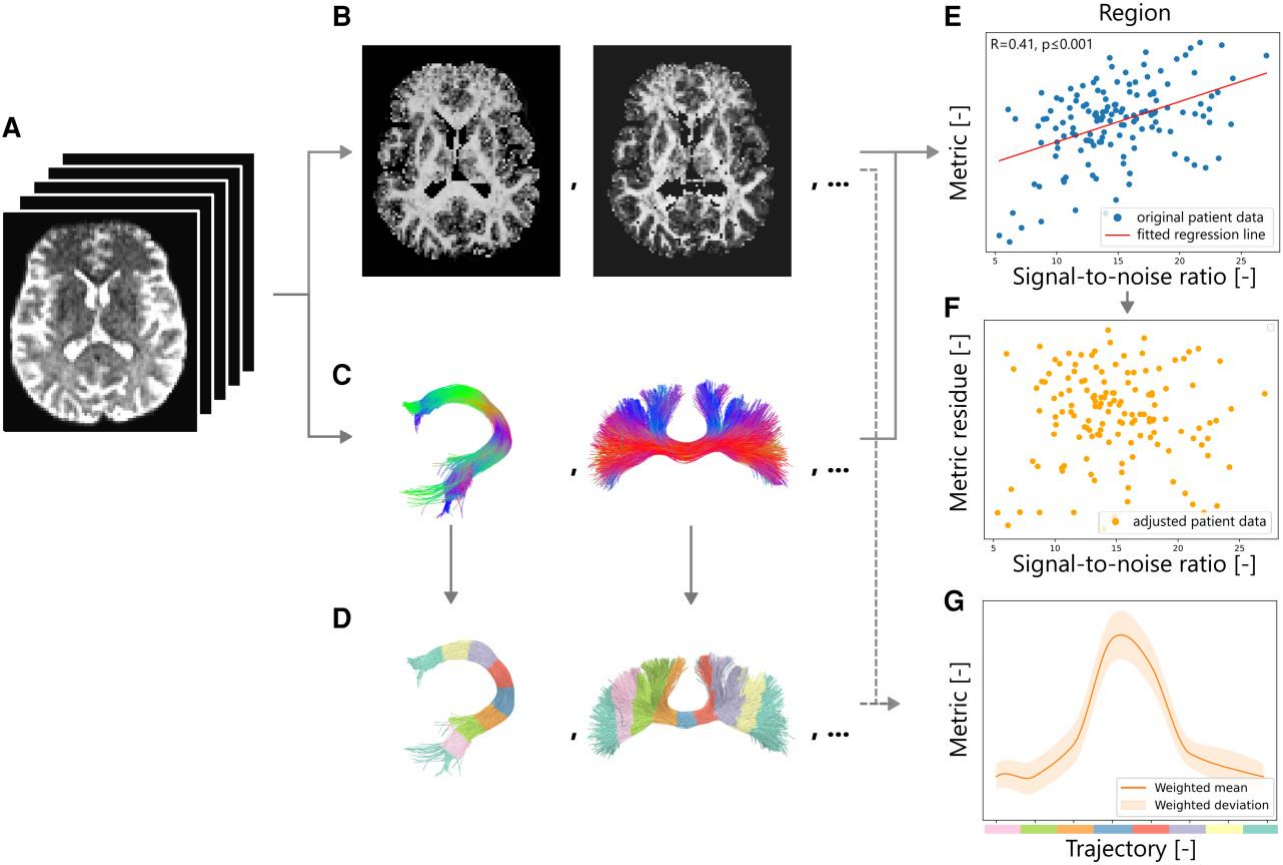
**Representation of the tract-specific analysis.** The preprocessed dMRI data (**A**) is processed with multi-fixel models to estimate microstructure metrics (**B**) and with tractography algorithms to generate the streamlines of interest (**C**). Both are then combined to provide an average metric per tract (**E**) for each participant (*n* = 119 scans) which is corrected for movement (**F**). The tracts are also divided into subsections along their pathway (**D**) to obtain the metric evolution along the tract trajectory (**G**). The tracts are colour-coded according to the orientation of the streamline segments: red (left–right), green (anterior–posterior) and blue (inferior–superior), with mixtures of the three primary colours indicating streamline segments oriented obliquely between these axes.

With the tracts of interest and microstructure estimates at our disposal, the next step consisted of computing tract-specific maps (Fig. 4B) and averages (Fig. 4E) for each metric in the tracts of interest (Fig. 4C). Unlike whole-brain maps, which combined fixel metrics based on their relative volume fraction, tract-specific maps leverage knowledge about the orientation of the tract of interest by weighting each fixel’s contribution according to its angular alignment with the tract’s main trajectory. The mean metric value per tract was obtained with the UNRAVEL framework using the angular weighting for the attribution fixel properties, and the streamline density weighted average to decrease the effect of stray streamlines.^34,53^

For each region, estimates of the tract-specific FA, AD, RD, MD and volume fraction of the diffusion tensors obtained with DIAMOND were reported. To provide more insight into the specific location of the differences between the two cohorts, the evolution of the metric values along the tract pathway (Fig. 4G) was calculated by partitioning the mean trajectory into eight sub-sections (Fig. 4D) using the methodology described in.^44^

Due to the implementation of gradient cycling on the MRI scanner, patient movement in the scanner introduced noise. Since metrics such as FA and others are susceptible to the influence of noise and motion,^54,55^ we opted to apply linear regression on each metric *M* to remove the influence of noise (Fig. 4E)


$$\hat{M}=\beta_{0}+\beta_{CNR}.X_{CNR}$$

$$\;M_{res}=M-\hat{M}$$

where *X*_CNR_ is the average contrast-to-noise ratio computed with FSL’s motion correction routine,^33^  *β*_0_ is the intercept, and *β_SNR_* is the regression slope. The outcome consisted of tract-specific metrics *M*_*res*_, enabling a comparison of differences unattributed to noise between the two cohorts (Fig. 4F).

The microstructural metrics of the AUD cohort and control cohort were compared with Welch’s *t*-tests to account for the unequal variances and sample sizes. To account for multiple comparisons, the Bonferroni correction was applied to the significance level.

To evaluate longitudinal effects, we fitted linear fixed-effects models including group (AUD, control) and time (E1, E2) as fixed factors. We did not include subject as a random intercept in a mixed-model because it prevented model estimation for some metrics (RD and MD) due to multicollinearity among tract predictors, while for other metrics (AD and FA) results from the fixed-effect models and mixed-effect models were highly consistent.

Additionally, to assess the associations between the average alcohol consumption (alcohol unit/day) and both the mean metric values at E1 and their changes between E1 and E2, linear regression models were fitted. Bonferroni correction was similarly applied to control for multiple testing in this analysis.

### Clinical associations

Self-report questionnaires of depression, obsession-compulsion, and anxiety were compared between subjects and controls at admission (E1), as well as within subjects between E1 and E2. Then, correlations between clinical scores and tract-specific microstructural metrics at E1 (FA, AD, RD and MD) as independent variables were computed to explore the link between dMRI metrics and behaviour. The same correlations were computed within the control group and at E2. Additionally, a linear mixed-effect regression model was conducted to predict depression levels based on group (subjects, controls), timepoint (E1, E2) and microstructural metrics across tracts (FA, AD, RD and MD), with subject included as a random intercept. A threshold of a *P*-value of 0.05 was set for significance in these analyses.

### Statistical analyses

The statistical methods applied in the different analytical steps are summarized below.

**Whole-brain analysis** To compare the AUD and control groups, a single-factor analysis of variance (ANOVA) corrected for age was performed. Statistical significance was set at *P* < 0.0001. Correction for multiple comparisons was applied using cluster-size thresholding.

**Tract-specific analysis** Group differences in microstructural metrics between the AUD and control cohorts were assessed using Welch’s *t*-tests to account for unequal variances and sample sizes. Multiple comparisons were corrected using the Bonferroni method, with the significance level set at *P* < 0.05. To examine associations between average alcohol consumption (alcohol units/day) and both the mean metric values at E1 and their changes between E1 and E2, linear regression models were fitted. Bonferroni correction was also applied to control for multiple testing in this analysis.

**Clinical associations analysis** Self-report questionnaire scores were compared between AUD participants and controls at admission (E1), and within the AUD group between E1 and E2. Correlations between clinical measures and tract-specific microstructural metrics at E1 were computed to investigate relationships between diffusion MRI metrics and behavioural outcomes. The same analyses were repeated for the control group and at E2. Additionally, a linear mixed-effect regression model was conducted to predict depression levels based on group, timepoint and microstructural metrics across tracts, with subject included as a random intercept. A significance threshold of *P* < 0.05 was used for these analyses.

## Results

### Clusters highlighted in the whole-brain analysis

The analysis of the whole-brain microstructural maps representing the volume fraction weighted metrics obtained with Eq. (1) identified several clusters of interest in the WM, which are displayed in Table 1. The bilateral tracts containing these clusters were the cingulum, fornix, corpus callosum, uncinate fasciculi, anterior thalamic radiations, and inferior longitudinal fasciculus. The dorsal part of the cingulum presented more clusters than the other sections. The clusters were present in different radiations and sections of the corpus callosum, namely, the forceps minor, forceps major and the isthmus.

**Table 1:** Clusters exhibiting significant differences between the AUD and control groups

	Voxel coordinates					
#	x	y	z	Volume [*mm*^3^]	*t*-value	Anatomical label
wMD						
1	−7	−33	36	30	−5.458	Cingulum (dorsal, L)
wRD						
1	28	−14	−26	48	−5.190	Fornix (temporal, R)
2	15	50	25	83	−5.747	Forceps minor
3	10	−11	39	33	−5.927	Cingulum (dorsal, R)
4	6	−18	37	34	−5.623	Cingulum (dorsal, R)
wAD						
1	35	8	−12	37	6.107	Uncinate fasciculus (R)
2	13	50	30	18	5.883	Forceps minor
3	10	33	13	349	6.447	Cingulum (dorsal, R)
4	7	−8	4	61	5.422	Anterior thalamic radiation (R)
5	−6	25	20	303	6.744	Cingulum (anterior, L)
6	−6	11	31	33	5.745	Cingulum (dorsal, L)
7	−24	−47	1	20	5.633	Forceps major
8	−24	22	−16	18	5.106	Uncinate fasciculus (L)
9	−32	12	−8	49	5.912	Uncinate fasciculus (L)
wFA						
1	52	−11	−35	42	5.633	Inferior longitudinal fasciculus (R)
2	45	−2	−35	54	6.413	Inferior longitudinal fasciculus (R)
3	38	−8	−13	115	6.039	Uncinate fasciculus (R)
4	34	10	−13	277	6.816	Uncinate fasciculus (R)
5	16	−45	22	76	5.126	Corpus callosum (isthmus)
6	13	14	33	91	6.321	Cingulum (dorsal, R)
7	8	30	20	199	6.319	Cingulum (dorsal, R)
8	−7	21	25	102	6.090	Cingulum (anterior, L)
9	−20	−54	15	91	6.033	Forceps major
10	−22	−25	−16	46	6.176	Cingulum (temporal, L)
11	−32	12	−8	59	5.950	Uncinate fasciculus (L)
12	−42	−5	−17	126	5.832	Uncinate fasciculus (L)

Voxel coordinates of the cluster centres in MNI space, volumes, *t*-values, as well as the corresponding anatomical labels (L: left, R: right), for each statistically significant cluster of the wMD, wRD, wAD and wFA metrics. All corresponding *P*-values for the clusters presented were below 0.0001.

Overall, the clusters were located mainly in the WM pathways connecting the frontal and temporal lobes, as well as the limbic system. The clusters found with the volume-weighted MD and RD (wMD and wRD) maps showed higher values in the AUD participants, while those found with the volume-weighted AD and FA (wAD and wFA) maps displayed lower values in the AUD cohort compared to the controls.

### Microstructure of the tracts of interest

**Figure 5:**
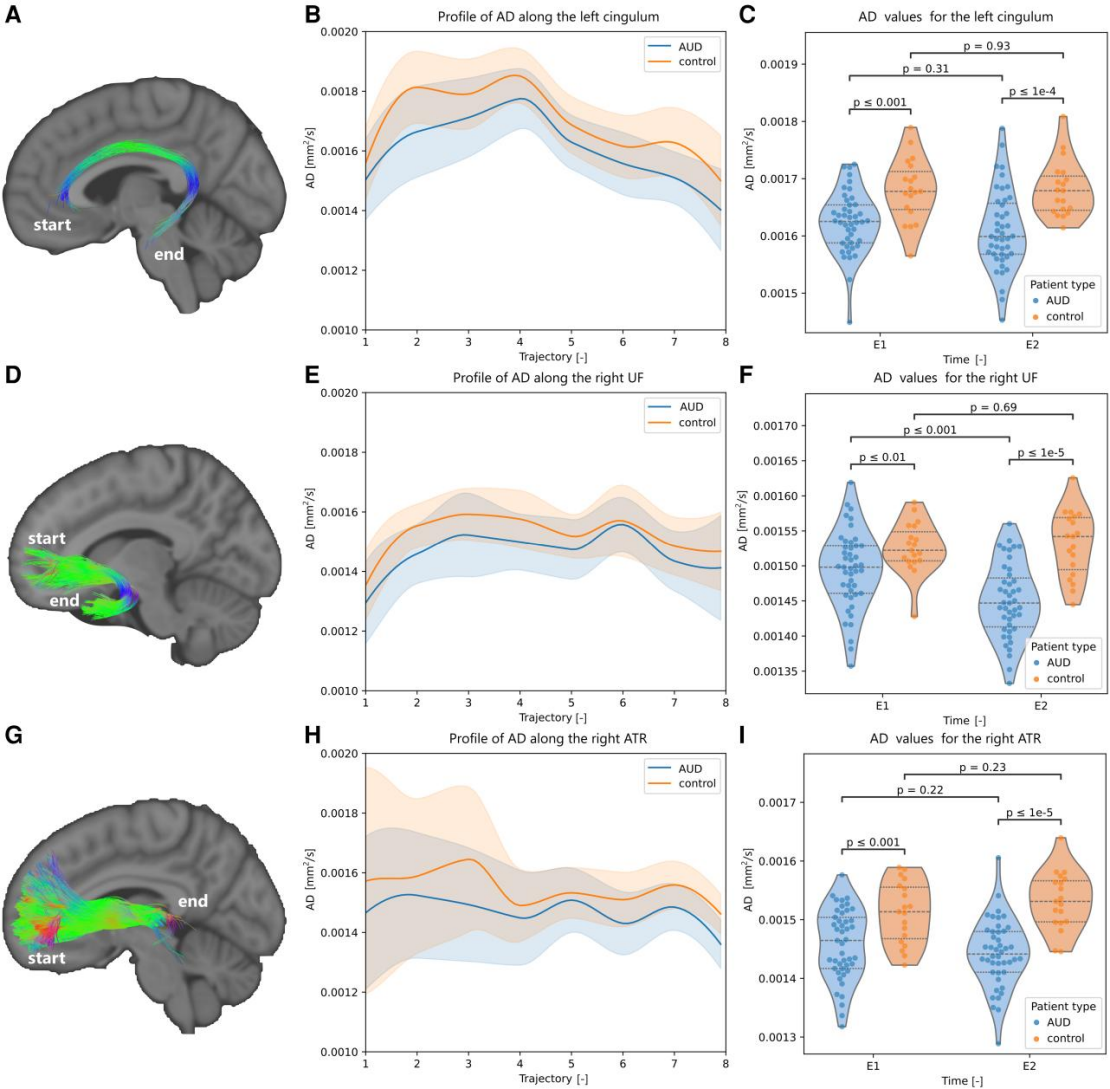
**Tract-specific microstructural differences across both populations (Part I).** Representation of the (**A**) cingulum, (**D**) uncinate fasciculus and (**G**) anterior thalamic radiations. The tracts are colour-coded according to the orientation of the streamline segments: red (left–right), green (anterior–posterior) and blue (inferior–superior), with mixtures of the three primary colours indicating streamline segments oriented obliquely between these axes. For each tract, the evolution of the statistically significant tract-specific axial diffusivity (AD) along the tract pathway at E1 (*n* = 43 AUD and 20 control participants) is displayed (**B, E, H**), with violin plots of the distributions of the microstructural metric for the AUD (blue) and control (orange) population, before (E1, *n* = 43 AUD and 20 control participants) and after (E2, *n* = 37 AUD and 19 control participants) an 18-day abstinence period (**C, F, I**). The *P*-values were obtained using Welch’s *t*-tests.

**Figure 6:**
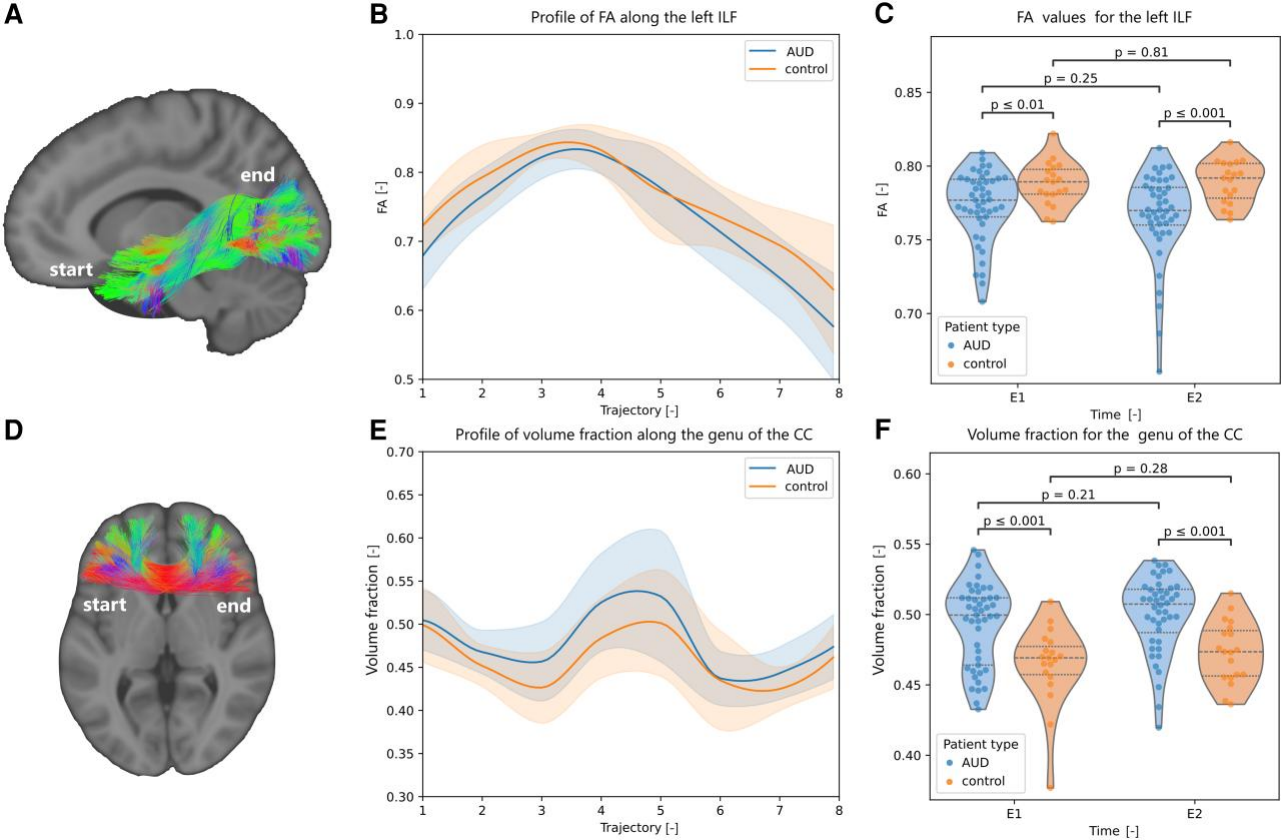
**Tract-specific microstructural differences across both populations (Part II).** Representation of the (**A**) inferior longitudinal fasciculus and (**D**) genu of the corpus callosum. The tracts are colour-coded according to the orientation of the streamline segments: red (left–right), green (anterior–posterior) and blue (inferior–superior), with mixtures of the three primary colours indicating streamline segments oriented obliquely between these axes. For each tract, the evolution of a statistically significant tract-specific metric (FA or volume fraction) along the tract pathway at E1 (*n* = 43 AUD and 20 control participants) is displayed (**B, E**), with violin plots of the distributions of the microstructural metric for the AUD (blue) and control (orange) population, before (E1, *n* = 43 AUD and 20 control participants) and after (E2, *n* = 37 AUD and 19 control participants) an 18-day abstinence period (**C, F**). The *P*-values were obtained using Welch’s t-tests.

The tract-specific metrics estimated for each of the tracts identified in the whole-brain analysis were compared between cohorts. The statistically significant *P*-values obtained from Welch’s t-tests, assessing differences between cohorts at each time point for each tract of interest, are presented in Figs 5 and 6. Additional graphs are depicted in the supplementary materials (Supplementary Fig. 1).

The tract-specific AD was significantly lower in the left cingulum (Fig. 5C), right and left uncinate fasciculus (Fig. 5F, Supplementary Fig. 1C), right and left anterior thalamic radiations (Fig. 5I, Supplementary Fig. 1E), left inferior longitudinal fasciculus (Supplementary Fig. 1H), and the genu of the corpus callosum (Supplementary Fig. 1I) for AUD participants compared to controls. Lower AD in these tracts may reflect axonal injury or altered axonal organization, consistent with reduced WM integrity in AUD. The streamlines passing through the genu of the corpus callosum in the tract-specific analysis correspond to the forceps minor, listed in Table 1.

The evolution of the metric along the tracts showed a global decrease in mean AD for participants with AUD in the left cingulum (Fig. 5A and B), with a decreased mean value throughout the tract. In contrast, the along-tract analysis in the uncinate fasciculus and anterior thalamic radiations (Fig. 5D, E, G and H) indicated that the primary differences between both cohorts were predominantly localized in the frontal and temporal segments, and near the basal ganglia, respectively. The temporal section of the anterior thalamic radiations was characterized by a higher standard deviation across participants in both cohorts.

The tract volume fraction was higher for participants with AUD in the left and right anterior thalamic radiations (Supplementary Fig. 1F and G), genu of the corpus callosum (Fig. 6F) and in the left uncinate fasciculus (Supplementary Fig. 1D). The tract-specific FA in the left and right cingulum (Supplementary Fig. 1A and B), and left inferior longitudinal fasciculus (Fig. 6C) was lower in participants with AUD. Reduced FA is generally interpreted as evidence of diminished WM integrity, likely due to axonal degeneration, or increased diffusivity perpendicular to axonal fibres. The main differences between control and AUD in the inferior longitudinal fasciculus were located in the temporal section and near the occipital cortex (Fig. 6A and B).

Although correlations between tract-specific mean metrics and average alcohol consumption revealed certain trends, these did not reach statistical significance after correction for multiple comparisons. The results of the linear regression analyses, including corresponding *R* and *P*-values prior to adjustment, are presented in the supplementary materials (Supplementary Fig. 2).

### Differences in clinical scores and tract correlations

Self-report questionnaires were compared between E1 and E2, and between AUD participants and controls in Table 2. At E1, AUD participants exhibited significantly higher scores for depression (BDI), anxiety (STAI), and obsessions-compulsions (OCDS) compared to controls. Among the AUD participants, there was a significant reduction in all clinical measures between E1 and E2, shown in Table 2.

**Table 2:** Comparison of behavioural scores between E1 and E2 in case subjects, and between case and controls at E1, for several questionnaires: Beck Depression Inventory (BDI) for depression, the State Anxiety Inventory (SAI) for anxiety, and the Obsessive Compulsive Drinking Scale (OCDS) for obsessions and compulsions related to drinking

	E1	vs E2			vs Controls		
	Mean	Mean	*t*-value	*P*-value	Mean	*t*-value	*P*-value
BDI	22.41	14.41	4.874	<0.001	5.42	8.136	<0.001
OCDS	14.65	8.27	5.567	<0.001	1.32	10.430	<0.001
SAI	44.57	38.84	3.455	<0.001	29.16	5.746	<0.001

Correlation between microstructural metrics and clinical measures were computed at E1 for AUD patients, using Pearson’s or Spearman’s test depending on the normality of the data distribution (see Table 3). Results were corrected for false discovery rate (FDR). Significant associations were primarily observed between depression and tract-specific FA and RD across the right anterior thalamic radiations, the isthmus of the corpus callosum and the right uncinate fasciculus. Specifically, depression showed a positive correlation with RD, and a negative correlation with FA. No significant correlations were found for anxiety and OCDS. Additionally, no region demonstrated consistent associations across all metrics. There were no corresponding correlations in the control group, nor were there any new significant correlations. To assess the persistence of these correlations following withdrawal, the analyses were repeated at E2. None of the previous correlations remained significant, and no new significant correlation appeared.

**Table 3:** Significant correlations between the tract-specific diffusion metrics and depression (BDI) scores in the AUD population at E1

	Correlation coefficient	*P*-value	B-H value
**FA** Right anterior thalamic radiations	Pearson (*r* = −0.455)	0.047	0.0100
Isthmus of the corpus callosum	Pearson (*r* = −0.514)	0.011	0.0033
Right uncinate fasciculus	Spearman (*ρ* = −0.463)	0.039	0.0067
**RD** Isthmus of the corpus callosum	Pearson (*r* = 0.477)	0.028	0.0033

The *P*-value and corresponding Benjamini–Hochberg (B–H) critical value are reported in the right-most columns.

Given that depression exhibited the strongest associations at E1, a linear regression model was computed to examine the relationship between depression scores and mean-centred FA measures across all tracts of interest. Group (patients vs. controls) and time (E1 vs. E2) were included as independent predictors (main effects only; no interaction term). Depression scores were significantly higher in patients compared to controls (*B* = −14.96, *t* = −5.391, *P* < 0.001), and significantly decreased over time (*B* = −8.31, *t* = −3.868, *P* < 0.001), indicating main effects of both group and time. Higher FA in the genu of the corpus callosum (*B* = 176.47, *t* = 2.078, *P* = 0.041) was associated with higher depression scores, while lower FA in the isthmus of the corpus callosum (*B* = −214.56, *t* = −2.071, *P* = 0.042), left fornix (*B* = −28.67, *t* = −2.515, *P* = 0.014), and right uncinate fasciculus (*B* = −127.75, *t* = −2.302, *P* = 0.024) were linked to higher depression scores. Multicollinearity diagnostics confirmed that all reported predictors had a variance inflation factor (VIF) below 10.

The model was also tested with mean-centred RD, AD and MD measures instead of FA as predictors. Higher RD in the isthmus of the corpus callosum (*B* = 267 333.36, *t* = 2.522, *P* = 0.014) and right fornix (*B* = 48 694.87, *t* = 2.830, *P* = 0.006) were associated with higher depression scores. Conversely, higher RD in the genu of the corpus callosum was linked to lower depression scores (*B* = −124 704.40, *t* = −2.025, *P* = 0.046). Higher AD in the posterior midbody of the corpus callosum was correlated with higher depression scores (*B* = 105 715.153, *t* = 2.056, *P* = 0.044), as well as higher MD in the right uncinate fasciculus (*B* = 154 440.111, *t* = 2.136, *P* = 0.036). Across all models, both group (patients vs. controls) and time (E1 vs. E2) remained significant predictors of depression scores (*P* < 0.001), with all VIF values below 10, indicating no multicollinearity concerns.

## Discussion

### Specific white matter pathways are affected by AUD

Our study leverages a precise and novel approach—whole-brain analysis using volume-weighted multi-fixel metrics—which enhances spatial sensitivity and specificity over traditional single-fixel analysis of dMRI data. The clusters identified with this approach predominantly overlapped with the WM pathways previously reported as clusters of convergent alterations in single-fixel analyses in AUD patients. Specifically, regions of the fornix, cingulum, uncinate fasciculus, anterior thalamic radiations, inferior longitudinal fasciculus and corpus callosum were highlighted in the whole-brain volume-weighted metric maps. Interestingly, our multi-fixel approach allowed us to uncover changes in regions that had not been identified with previous single-fixel analysis. The primary differences from the meta-analysis by Spindler *et al*.^20^ included the presence of clusters of interest in the uncinate fasciculus, anterior thalamic radiations and inferior longitudinal fasciculus and the absence of a cluster in the internal capsule. However, it should be noted that the anterior thalamic radiations originate near the basal ganglia and pass through the internal capsule, a region populated with several neural pathways. In a single-fixel analysis, this overlap may lead to ambiguity, with changes in the anterior thalamic radiations being potentially confounded with those of other pathways in the internal capsule. This effect is mitigated in multi-fixel models, which offer improved specificity. Furthermore, the uncinate fasciculus was mentioned as a tract of interest in previous studies on alcohol-induced damage in early abstinence.^17^

All examined regions showed significant differences between the AUD and control cohorts in the tract-based analysis, except for the fornix and the forceps major, where observed differences did not survive correction for multiple comparisons.

The microstructural differences between the AUD and control cohorts persisted following the supervised abstinence period. This finding aligns with and extends previous studies, which reported sustained microstructural alterations in the fornix and corpus callosum during early abstinence.^17,56^ Longitudinal studies following recovering AUD patients over several years showed improvement in brain fibre tract integrity reflective of a fibre reorganization and remodelling rather than a *restitutio ad integrum.*^57^ Our results indicate that this lack of recovery is evident across all examined WM regions, suggesting a widespread and persistent impact of excessive alcohol use on brain microstructure.

### Presence of global and localized differences on tracts

Our approach of labelling the neural pathways associated with each cluster and conducting an along-tract analysis along those pathways enabled detailed comparisons of metric profiles between the AUD and control cohorts. This approach revealed both global and localized alterations in the tract-specific microstructure of the affected WM pathways. Certain pathways, such as the cingulum, exhibited widespread metric alterations along their entire length, with more pronounced differences observed in the dorsal section. The dorsal cingulum is a key conduit for executive control, emotion regulation, and pain processing.^46^ Thus, heavier microstructural damage in the dorsal segment could contribute to the well-documented executive dysfunction and emotional dysregulation in AUD. Another study found that abstinent alcoholics had lower FA in frontal and limbic tracts (including the dorsal cingulum) relative to controls, and these FA reductions were associated with poorer executive function.^13^ In contrast, other tracts, such as the anterior thalamic radiations, displayed differences only in specific segments, in this case near the internal capsule. Such findings illustrate that AUD-related WM alterations can be both diffuse and tract-segment specific, potentially depending on regional fibre composition or localized susceptibility to ethanol’s neurotoxic effects. Future work correlating these segmental changes with domain-specific cognitive measures could further elucidate their functional importance.

### Microstructural alterations in affected tracts

The affected tracts predominantly exhibited a reduction in tract-specific AD, a decrease in tract-specific FA, or an increase in fibre fraction compared to controls. The differences in tract-specific MD and RD were less pronounced, suggesting that the commonly reported FA reduction in affected regions^17,56^ may be primarily driven by a decrease in AD rather than an increase in RD.

However, the underlying biological mechanisms and histological processes reflected by these microstructural alterations remain unclear. Although dMRI metrics are sensitive to biological changes, they lack specificity for different biological processes.^58^ Despite this, their significance can be hypothesized from common interpretations and post-mortem studies. The observed decrease in AD or FA may indicate axonal degeneration.^59^ Since compartment volume fractions sum up to unity, the increase in volume fraction could result from a reduction in the volume of crossing fibres or the volume attributed to isotropic diffusivity, such as cerebrospinal fluid or extracellular bodies. Further histological studies are required to investigate the precise biological processes underlying the observed changes.

### Link with clinical measures and potential effects on brain function

Clinical scores of depression, obsession-compulsion and anxiety were significantly higher in the AUD group than in controls, and declined significantly from E1 to E2 among the AUD patients. This study explored how changes in WM microstructure could correlate with these clinical differences between groups and timepoints.

The most consistent findings in the AUD patient group at E1 were observed in the correlations between tract-specific microstructural indices and depression scores. Patterns indicative of axonal degeneration, characterized by reductions in FA, were associated with higher depression scores in several regions, including the corpus callosum, the right uncinate fasciculus, and the right anterior thalamic radiations. Demyelination patterns, often associated with decreased FA and increased RD, correlated with high depression scores in the isthmus of the CC.^59^ A previous study in depressive subjects showed an inverse correlation between FA in the corpus callosum and peripheral inflammation.^60^ The neuroinflammation observed at the beginning of alcohol withdrawal in AUD patients,^61,62^ when BDI scores correlate with serum interleukin 6 (IL-6) levels, could underlie the observed relationship between structure and depression in our cohort.

These findings were not observed in the control group or in the AUD group at E2, suggesting that the observed correlations between microstructural integrity and depression scores are specific to active AUD and confined to a very early stage of withdrawal. Efforts to produce a comprehensive model predicting depression across subjects and timepoints showed some interesting leads. In the isthmus of the corpus callosum and fornix, lower FA and higher RD were predictive of higher depression score. Additionally, a pattern of increased FA and lower RD in the genu of the corpus callosum was found to correlate with higher depression scores. These findings suggest a regionally specific pattern within the corpus callosum in relation to depression. The WM fibres of the genu connect the bilateral prefrontal and orbitofrontal cortices, which are related to decision-making, attention, reward processing, and emotion regulation.^63^ All these functions are essential to mood regulation; a pattern of high FA and low RD correlating to high depression in these areas appears counterintuitive but could be linked to compensatory mechanisms in the context of acute withdrawal and clinical improvement. On the other hand, the isthmus mostly contains primary motor, somatosensory, and auditory fibres of large diameter (3–5 μm)^64^ and may be more vulnerable to demyelination and neuroinflammatory processes during withdrawal.

The precise functional implications of the observed microstructural changes remain difficult to determine as we did not directly assess several cognitive and behavioural outcomes. However, examining the presumed functions of these tracts can provide insight into the cognitive and clinical processes potentially affected by these alterations.

The impact of excessive alcohol consumption on the microstructure of the fornix might have consequences on memory formation and cognitive flexibility, insofar as the fornix pathway conveys mnemonic representations to brain structures involved in guiding motivated behaviour.^45^ The alterations caused by AUD in the dorsal cingulum could potentially affect the regulation of executive control, the formation of emotion, the appraisal of pain and the reinforcement of behaviour reducing pain.^46^ The decreased AD in the uncinate fasciculus might impair the reversal learning performance of chronic alcohol consumers, in keeping with prior results showing a significant negative relationship between the reversal learning performance and the AD of the uncinate.^65^ Additionally, the uncinate fasciculus connects key regions of the cortico-limbic circuit—namely the amygdala and ventral prefrontal cortex—and has been associated with emotion regulation, with microstructural alterations already reported in patients with major depressive disorder.^66^ The anterior thalamic radiations are part of the frontostriatal circuit, which is thought to mediate inhibitory control. Disruptions or microstructural changes in this circuit may result in impulsive behaviour, impairments in response inhibition, motivation, and inability to maintain sobriety.^67,68^ The inferior longitudinal fasciculus is involved in the processing and modulation of visual signals, which affects visually guided decisions and behaviours.^51^ Moreover, AUD may influence the regulation of emotions through its effects on the corpus callosum, which plays an important role in the communication of perceptual, cognitive, and learned information between hemispheres.^52^ More specifically, neural fibres traversing the genu of the corpus callosum, also known as the forceps minor, which connect to the prefrontal cortex, are known to mediate structural connectivity among central executive, salience and default mode networks.^12^ Previous DTI studies have linked the forceps minor’s microstructure to cognitive reappraisal, a central process of emotion regulation.^69^ While these interpretations remain speculative, they are informed by the known functions of these white matter pathways and align with prior findings in clinical populations.

Altogether, the only symptoms that proved to be related to microstructure changes were depressive symptoms, supporting the notion that WM alterations could play a role in the so-called ‘dark side’ of AUD, with consequences for the risk of relapse due to unresolved depressive mood. Changes observed in the corpus callosum could be related to the decreases in interhemispheric connectivity that have been posited to play a role in the expression of depressive symptoms.^63^

### Limitations

Our study relies on two well-characterized and carefully matched groups, studied at two precise timepoints during their early abstinence. However, the 18-day abstinence period may have been insufficient to capture the full extent of microstructural changes or their recovery. Prior evidence suggests that alterations during early abstinence typically occur over a 2- to 6-week interval.^56^ However, ensuring patient abstinence over longer intervals presents practical challenges. Notably, our in-patient study setting ensured a controlled environment, minimizing variability due to uncontrolled alcohol intake and improving reliability in capturing early abstinence effects. Additionally, the relatively small sample size calls for replication in larger cohorts to validate the findings. However, our approach replicated several clusters of interest reported in prior studies, lending further credibility to the results presented.

Furthermore, the analysis treated the cingulum as a single continuous bundle, despite evidence that it can be subdivided into two, three, or five distinct regions with unique connections and functions.^46,70^

We did not include smoking status as a covariate in our analyses because only binary (yes/no) smoking data were available for the patient group, and no smoking data were collected for controls. While prior large-scale studies have shown that smoking and alcohol use tend to affect white matter microstructure in similar ways^71^—both associated with lower fractional anisotropy and higher mean diffusivity—thus reducing the likelihood that smoking alone introduces a confounding pattern, the possibility remains that smoking could moderate the trajectory of white matter recovery during abstinence. Notably, Gazdzinski *et al*. reported that smoking status influenced recovery patterns in abstinent AUD patients: while non-smokers showed improvements in white matter microstructure over one month, smokers exhibited only macrostructural volume gains without corresponding microstructural recovery.^72^ Future studies incorporating detailed smoking histories and longitudinal imaging over longer periods are warranted to clarify the interactive effects of alcohol and tobacco use on white matter recovery.

## Conclusion

The results of our study highlight the potential of leveraging multi-shell data, multi-fixel microstructural modelling and tract-specific analyses to refine the description of white matter alterations associated with chronic AUD. The present findings confirm the existence of microstructural alterations along different major white matter bundles in subjects with AUD. While these results partially overlap with previous findings, our technique afforded greater spatial precision in mapping alterations along the trajectory of specific white matter bundles—a level of detail that, to the best of our knowledge, has not been previously achieved, despite the well-established vulnerability of white matter in AUD. The impact of alcohol dependence on the microstructure of white matter tracts associated with these clusters is demonstrated to remain into early abstinence, revealing both localized and widespread alterations. This persistence of microstructural alterations during almost three weeks of abstinence, despite rapid improvements in clinical measures, points to medium to long-lasting effects of excessive alcohol consumption on these structures. Among the self-report questionnaires, these alterations in white matter structures correlated mainly with higher depression scores, where depression could sustain a pattern of excessive intake. Microstructural metrics in certain structures, such as the isthmus of the corpus callosum, exhibited higher correlations with clinical scores in the AUD cohort, whereas other structures, including the cingulum, uncinate fasciculus, and anterior thalamic radiations, displayed more pronounced microstructural differences between AUD and control groups. Overall, these findings emphasize the intricate relationship between white matter tract function, microstructural integrity, and patient behaviour in AUD. Integrating these observations with other modalities could unveil new biomarkers, paving the way for the development of personalized medicine with practical outcomes benefiting people affected by AUD.

## Supplementary Material

fcag018_Supplementary_Data

## Data Availability

The dMRI data was obtained in the Brussels Saint-Luc University Hospital, Belgium. The raw data are not publicly available due to privacy issues of clinical data. The mean values for the tract-specific analyses and the code used to generate the plots in the figures are available at: https://github.com/DelinteNicolas/AUD_microstructure.
