# Supplementary material for: White matter microstructure alterations from alcohol use disorder persist into early abstinence
Source: Brain Commun. 2026 Jan 20;8(1):fcag018. doi: 10.1093/braincomms/fcag018 (PMC12870131; doi:10.1093/braincomms/fcag018)
Supplement: fcag018_Supplementary_Data [file fcag018_supplementary_data.pdf]

A Supplementary material

	**AUD cohort** ($n = 37$)	**Control cohort** ($n = 19$)
**Age** (years)	47.08 ± 10.24	50.32 ± 14.81
**Sex**		
Male	29	12
Female	8	7
**Smoking Status**		
Smoker	22	Not reported
Nonsmoker	15	Not reported
**Alcohol consumption** (units/day)	15.92 ± 8.75	0

Supplementary Table 1: Demographic table of the final sample of 37 AUD participants and 19 controls.

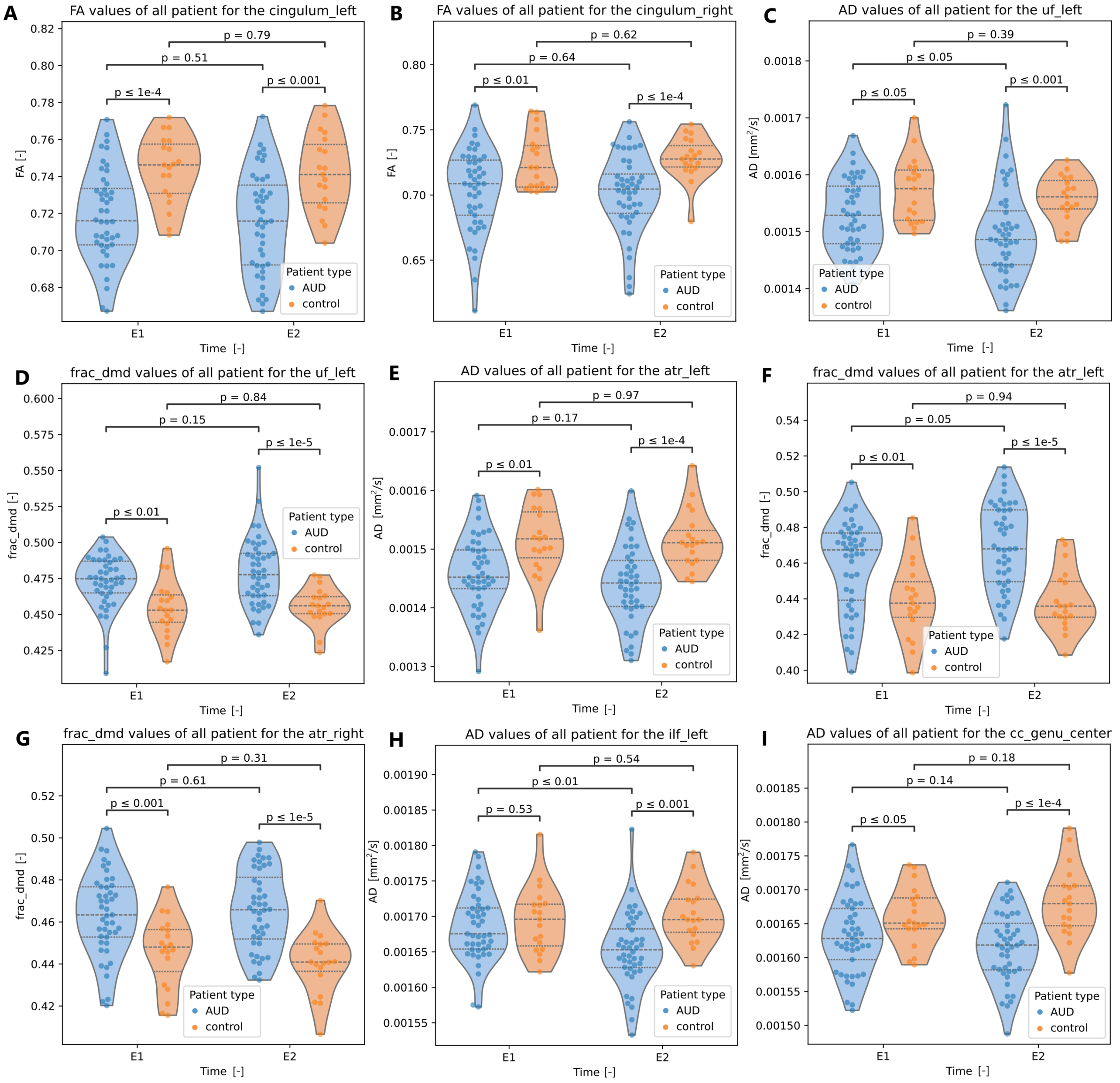

Supplementary Figure 1: Additional violin plots of the distribution of the microstructural metrics of the (**A, B**) cingulum, (**C, D**) uncinate fasciculus, (**E, F, G**) anterior thalamic radiations, (**H**) left inferior longitudinal fasciculus and (**I**) genu of the corpus callosum, for the AUD (blue) and control (orange) population, before (E1, n = 43 AUD and 20 control participants) and after (E2, n = 37 AUD and 19 control participants) an 18-day period of abstinence for the AUD group. The represented metrics include the fractional anisotropy (FA, in **A, B**), the axial diffusivity (AD, in **C, E, H, I**) and the volume fraction of the fiber population aligned with the tract of interest (*frac_dmd*, in **D, F, G**). Each data point represents an independent patient. The p-values were obtained using Welch's t-tests.

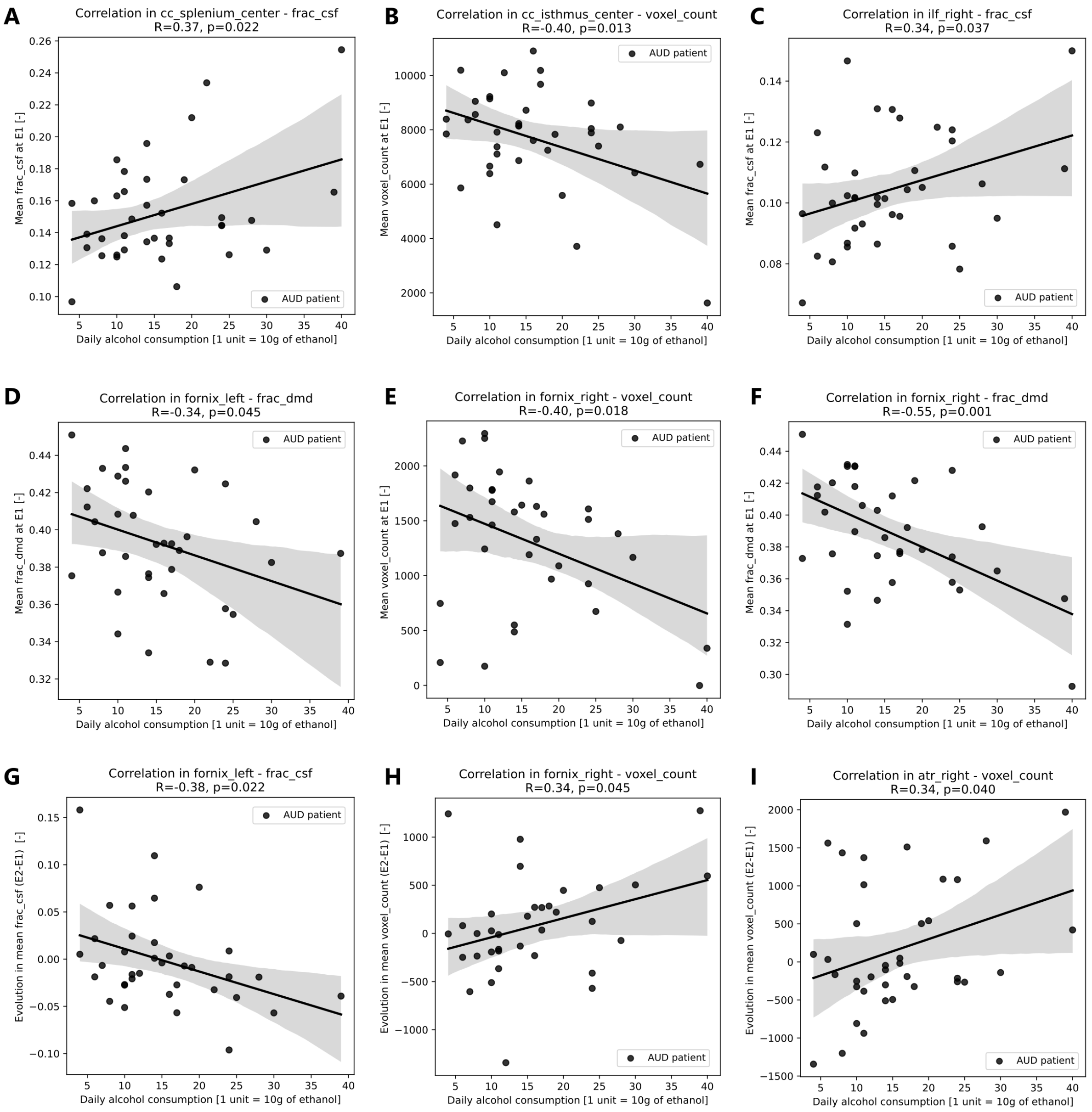

Supplementary Figure 2: Plots of the linear regression model illustrating the relationship between alcohol consumption and (**A , B, C, D, E, F**) the mean metric values at E1 (n = 43 AUD participants), and (**G, H, I**) the changes in these values over the 18-day period for the AUD population (n = 37 AUD participants). The represented metrics include the volume fraction of the isotropic compartment (*frac_csf*, in **A, C, G**), the volume fraction of the fiber population aligned with the tract of interest (*frac_dmd*, in **D, F**) and the number of voxels containing streamlines of the corresponding tract (*voxel_count*, in **B, E, H, I**). A 95% confidence interval for the regression estimate is depicted in grey around the regression line.
